# Effects of ranolazine on wild-type and mutant hNa_v_1.7 channels and on DRG neuron excitability

**DOI:** 10.1186/1744-8069-6-35

**Published:** 2010-06-08

**Authors:** Mark Estacion, Stephen G Waxman, Sulayman D Dib-Hajj

**Affiliations:** 1 Department of Neurology, Yale University School of Medicine, New Haven, CT 06510, USA; 2 Center for Neuroscience and Regeneration Research, Yale University School of Medicine, New Haven, CT, 06510 USA; 3 Rehabilitation Research Center, Veterans Affairs Connecticut Healthcare System, West Haven, CT, 06516, USA

## Abstract

**Background:**

A direct role of sodium channels in pain has recently been confirmed by establishing a monogenic link between *SCN9A*, the gene which encodes sodium channel Na_v_1.7, and pain disorders in humans, with gain-of-function mutations causing severe pain syndromes, and loss-of-function mutations causing congenital indifference to pain. Expression of sodium channel Na_v_1.8 in DRG neurons has also been shown to be essential for the manifestation of mutant Na_v_1.7-induced neuronal hyperexcitability. These findings have confirmed key roles of Na_v_1.7 and Na_v_1.8 in pain and identify these channels as novel targets for pain therapeutic development. Ranolazine preferentially blocks cardiac late sodium currents at concentrations that do not significantly reduce peak sodium current. Ranolazine also blocks wild-type Na_v_1.7 and Na_v_1.8 channels in a use-dependent manner. However, ranolazine's effects on gain-of-function mutations of Na_v_1.7 and on DRG neuron excitability have not been investigated. We used voltage- and current-clamp recordings to evaluate the hypothesis that ranolazine may be effective in regulating Na_v_1.7-induced DRG neuron hyperexcitability.

**Results:**

We show that ranolazine produces comparable block of peak and ramp currents of wild-type Na_v_1.7 and mutant Na_v_1.7 channels linked to Inherited Erythromelalgia and Paroxysmal Extreme Pain Disorder. We also show that ranolazine, at a clinically-relevant concentration, blocks high-frequency firing of DRG neurons expressing wild-type but not mutant channels.

**Conclusions:**

Our data suggest that ranalozine can attenuate hyperexcitability of DRG neurons over-expressing wild-type Nav1.7 channels, as occurs in acquired neuropathic and inflammatory pain, and thus merits further study as an alternative to existing non-selective sodium channel blockers.

## Background

There is substantial evidence for a critical role of sodium channels in acquired and inherited painful neuropathies, and non-selective sodium channel blockers are among first-line treatment for neuropathic and inflammatory pain, although they result in significant side effects which limits their clinical use [1]. The Nav1.7 sodium channel is preferentially expressed in sensory and sympathetic neurons and has been directly linked to painful disorders in humans; genetic studies have identified gain-of-function missense mutations within *SCN9A*, the sodium channel gene that encodes Na_v_1.7, in patients with inherited erythromelalgia (IEM), and a different set of gain-of-function missense mutations has been found in patients with paroxysmal extreme pain disorder (PEPD) [2,3]. Recently, loss-of-function mutations in Na_v_1.7 have been identified in individuals with congenital and complete inability to experience pain [2,3]. These studies provide compelling and complementary evidence for the role of this channel in pain signaling, and thus it has been considered to be a target for drug development.

Ranolazine is an anti-anginal drug which has been shown to preferentially block cardiac late (persistent) sodium current at concentrations that do not inhibit the peak transient current [4-6]. Ranolazine shortens the action potential duration in cardiac myocytes from mice with a long Q-T interval (LQT) Na_v_1.5 knock-in mutation but not from wild type (WT) mice [6]. Ranolazine acts as an open and inactivated-state blocker [7-9], its binding site overlaps with the local anesthetic receptor in domain 4/transmembrane segment 6 (DIV/S6) of voltage-gated sodium channels [6,8], and ranolazine appears to be a more effective anti-anginal agent compared to lidocaine [10]. Ranolazine has been shown to inhibit WT Na_v_1.7 and Na_v_1.8 in a use-dependent manner [8,9], and thus might be useful for treatment of hyperexcitability disorders of sensory systems, for example neuropathic and inflammatory pain caused by up-regulated expression of Na_v_1.7 [11,12].

We used voltage-clamp recordings to study the block of WT and IEM- and PEPD-related Na_v_1.7 mutations by ranolazine, and show a comparable block of peak and ramp currents of WT and mutant Na_v_1.7 channels. We also used current-clamp recordings to study firing of dorsal root ganglion (DRG) neurons transfected with WT and mutant Na_v_1.7 channels and show that ranolazine, at a clinically-relevant concentration, blocks high-frequency firing of DRG neurons expressing WT but not mutant Na_v_1.7 channels. These data are discussed in the context of using ranolazine for treatment of pain disorders.

## Results

**Figure 1:**
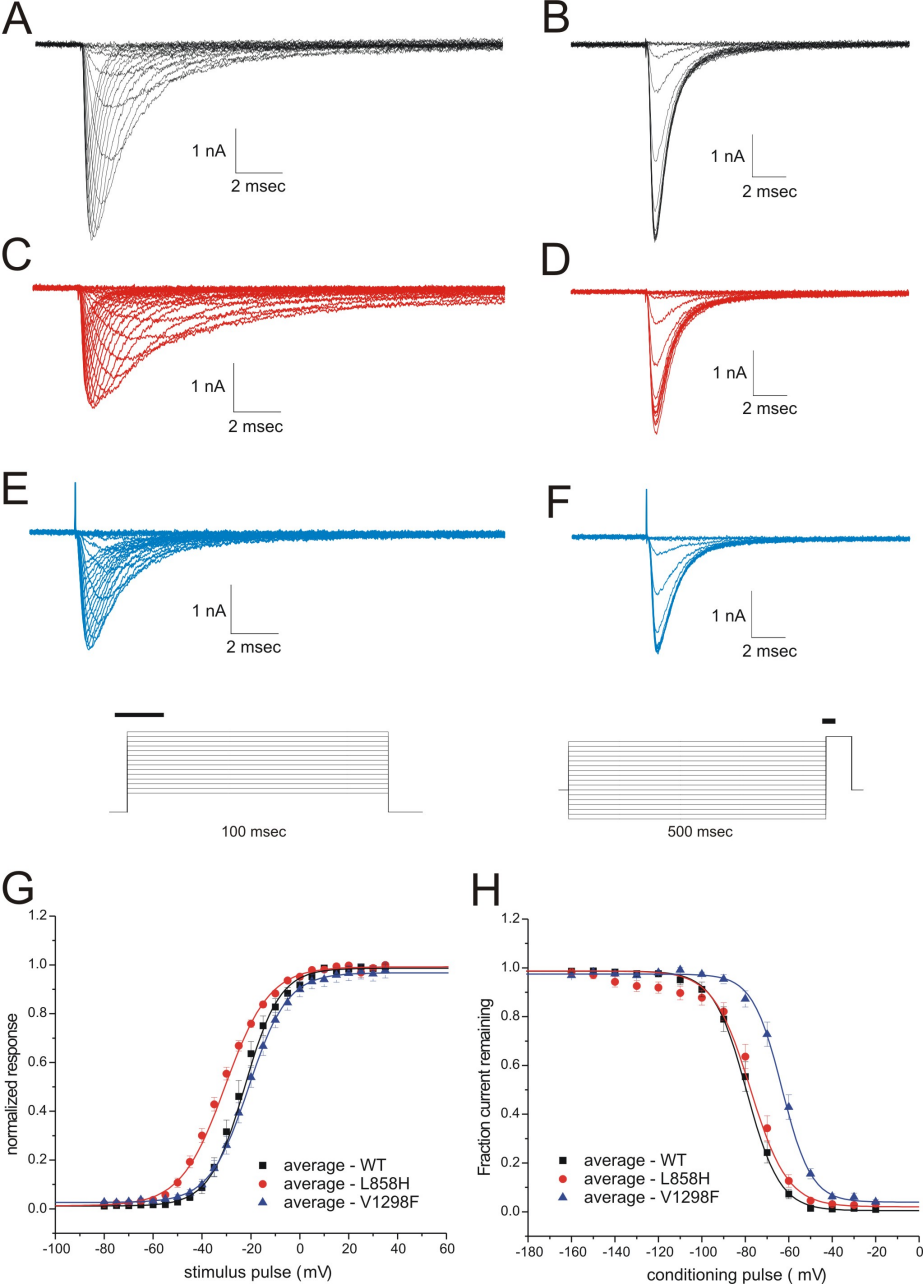
**Voltage-dependence of WT and the L858H and V1298F mutant channels**. (A) Superimposed activation traces recorded from a representative HEK + hNav1.7r-WT expressing cell. (Average peak current is -4.2 ± 0.7 nA, n = 12) (B) Superimposed fast-inactivation traces recorded from the same cell as in (A). (C) Superimposed activation traces recorded from a representative HEK + hNav1.7r-L858H expressing cell. (Average peak current is -2.3 ± 0.3 nA, n = 12). (D) Superimposed fast-inactivation traces recorded from the same cell as in (C). (E) Superimposed activation traces recorded from a representative HEK + hNav1.7r-V1298F expressing cell (Average peak current is -1.9 ± 0.3 nA, n = 18). (F) Superimposed fast-inactivation traces from the same cell as in (E). Insets illustrate the voltage pulse protocols for activation and fast-inactivation. The bold bars indicate the portion of the data sweeps displayed in this figure. (G) Normalized conductance-voltage (G-V) curves are constructed from the averages of individual HEK 293 cells expressing WT (black squares, n = 12), the IEM mutation L858H (red circles, n = 12), or the PEPD mutation V1298F (blue triangles, n = 18). (H) Normalized fast-inactivation curves are constructed from the averages of the same cells as in panel G.

To verify the typical biophysical signatures in HEK 293 cells for the hNa_v_1.7 mutant channels, the voltage-dependence of activation and fast-inactivation were determined for the IEM mutant L858H and the PEPD mutant V1298F and compared to WT channels. IEM mutations typically result in a hyperpolarized shift of the voltage-dependence of activation making the mutant channels easier to open in response to small depolarizations. Consistent with this, the V_1/2 _of activation for the L858H mutant channel (V_1/2 _= - 31.9 ± 1.2 mV, k = 9.2 ± 0.3; n = 11) was significantly (p < 0.001) shifted 8 mV in the hyperpolarized direction (Figure 1G) compared to WT channels (V_1/2 _= -23.8 ± 1.7 mV, k = 6.9 ± 0.5; n = 12). The V_1/2 _of activation for the PEPD mutation V1298F (V_1/2 _= -21.6 ± 1.4 mV, k = 7.4 ± 0.4; n = 18) was not significantly different from WT channels. In contrast, the biophysical signature for PEPD mutations is a depolarizing shift of the voltage-dependence of fast-inactivation predicting a greater availability of channels to open under conditions of sustained depolarizations. As expected, the V_1/2 _of fast-inactivation for the V1298F mutant (V_1/2 _= -63.3 ± 1.7 mV, k = 6.0 ± 0.3; n = 18) was significantly (p < 0.001) shifted 15.7 mV in the depolarized direction (Figure 1H) compared to WT channels (V_1/2 _= -79.0 ± 2.1 mV, k = 6.8 ± 0.4; n = 12). The fast-inactivation V_1/2 _for the IEM mutation L858H (V_1/2 _= -75.9 ± 1.9 mV, k = 8.5 ± 0.9; n = 11) was not significantly different from WT.

**Figure 2:**
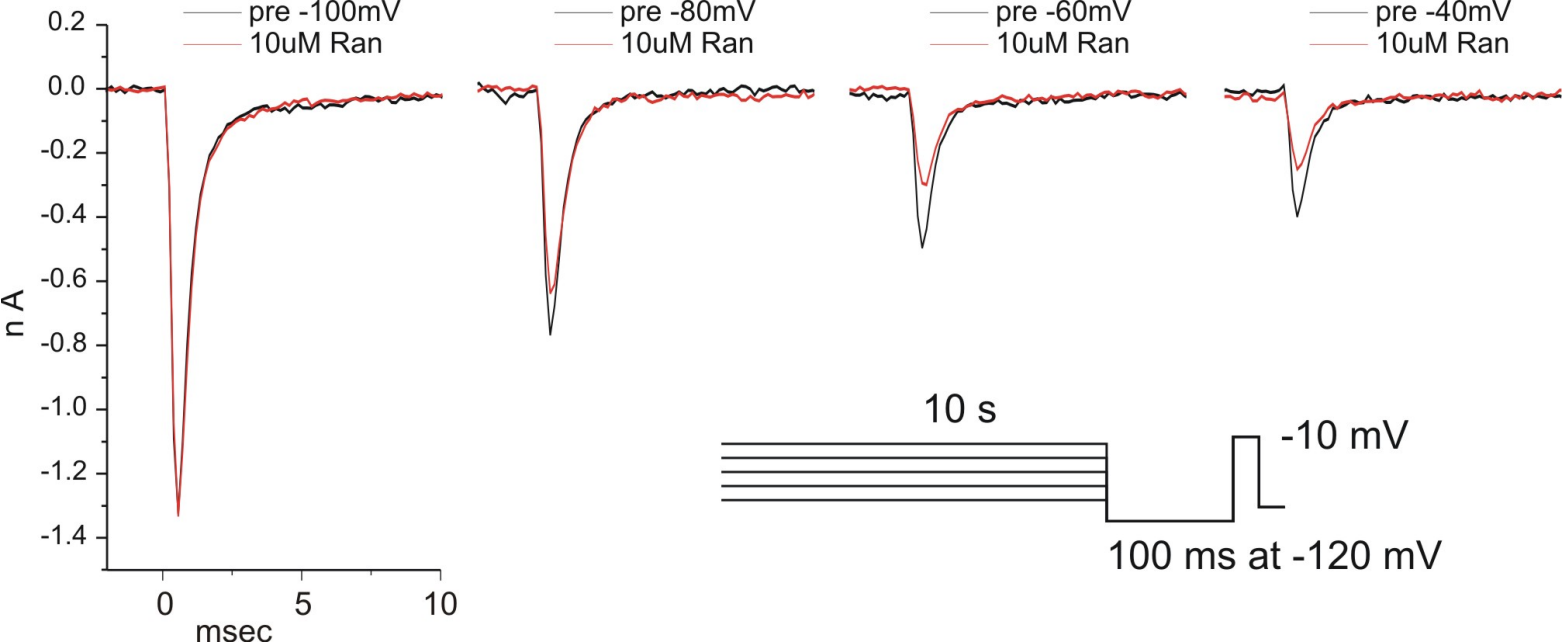
**Voltage-dependence of ranolazine block: Example traces**. The voltage-dependence of ranolazine block was determined by utilizing a ten-second conditioning pulse protocol as described in Methods. After the protocol was performed once (black traces), the cells were exposed to a single concentration of ranolazine and then the protocol was repeated (red traces). These data traces were obtained from an HEK + hNav1.7r-L858H expressing cell treated with 10 μM Ranolazine. The fraction of ranolazine block was determined at each conditioning potential by dividing the peak current in the presence of ranolazine by the baseline peak current.

**Figure 3:**
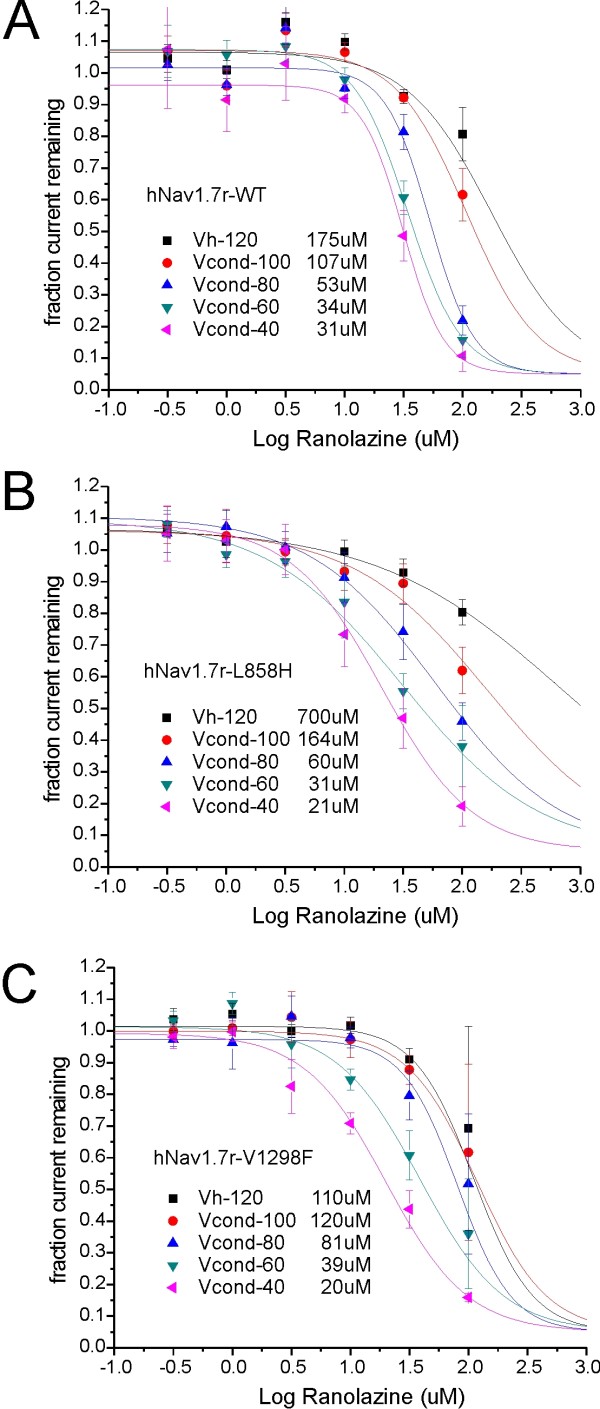
**Voltage-dependence of ranolazine block of hNav1**.7r-WT and L858H and V1298F mutants: Dose-response curve fits. The extent of ranolazine block for each cell was determined for conditioning potentials ranging from the holding potential of -120 mV (resting block) to Vcond of -40 mV (inactivated block). Each cell was exposed to only one concentration of ranolazine. The dose-response curve for each conditioning potential (see legend symbols) was obtained by fitting a single-site binding curve to the average current block (3-9 cells/point).

Ranolazine has recently been shown to block WT Na_v_1.7 and Na_v_1.8 channels [8,9] in a voltage-dependent manner, indicating higher affinity against inactivated channels. We examined whether either the L858H IEM mutation or the V1298F PEPD mutation show a differential sensitivity to ranolazine block at a holding potential (Vhold) of -120 mV or after 10-sec conditioning depolarizing potentials of -100 mV, -80 mV, -60 mV or -40 mV, compared to WT channels. To minimize the effect of slow changes in channel properties over time after achieving whole-cell dialysis, each cell was exposed to only one concentration of ranolazine. The fractional block to ranolazine at the various conditioning potentials was obtained by dividing the peak response to ranolazine by the peak response obtained during the baseline period as illustrated in figure 2. The dose-response dataset for a given conditioning potential was obtained by averaging 3-6 independent cells for each ranolazine concentration. As shown in figure 3A, the IC_50 _values for WT channels, derived by fitting a dose-response curve to the average block over six concentrations of ranolazine spanning the range of 0.3 μM to 100 μM, was voltage-dependent. The block was weakest for resting channels (IC_50 _= 175 μM at Vhold = -120 mV), and became stronger with increasing depolarizing conditioning potentials (IC_50 _= 34 μM at Vcond = -60 mV). For L858H mutant channels, our data indicate (Figure 3B) that ranolazine block is less effective for resting channels (IC_50 _= 700 μM at Vhold = -120 mV) but the increase with depolarization still occurs resulting in similar block as for WT (IC_50 _= 31 μM at Vcond = -60 mV). For the V1298F mutant channels (Figure 3C), the IC_50 _for ranolazine block is similar to WT at both resting (IC_50 _= 110 μM at Vhold = -120 mV) as well as depolarized (IC_50 _= 39 μM at Vcond = -60 mV) potentials. The parameters of the fitted dose-response curves for WT, L858H and V1298F channels are compiled in Table 1. The *fit comparison *statistic in Origin reports that the IC_50 _values at depolarized Vcond potentials -60 mV and -40 mV were significantly different than the IC_50 _for resting (Vhold -120 mV) values comparing within WT, L858H and V1298F data. This same statistic comparing between WT and either L858H or V1298F reports no significantly enhanced block by ranolazine at either resting (Vhold -120 mV) or depolarized (Vcond -60 mV or -40 mV) voltages.

**Table 1:** Summary of voltage-dependent ranolazine block

	WT	L858H	V1298F
Vhold = -120 mV	175 μM, R = 0.44	700 μM, R = 0.96	110 μM, R = 0.55

Vcond = -100 mV	107 μM, R = 0.79	164 μM, R = 0.93	120 μM, R = 0.83

Vcond = -80 mV	53 μM, R = 0.92	60 μM, R = 0.98	81 μM, R = 0.61

Vcond = -60 mV	34 μM, R = 0.99	31 μM, R = 0.97	39 μM, R = 0.74

Vcond = -40 mV	31 μM, R = 0.99	21 μM, R = 0.99	20 μM, R = 0.99

**Figure 4:**
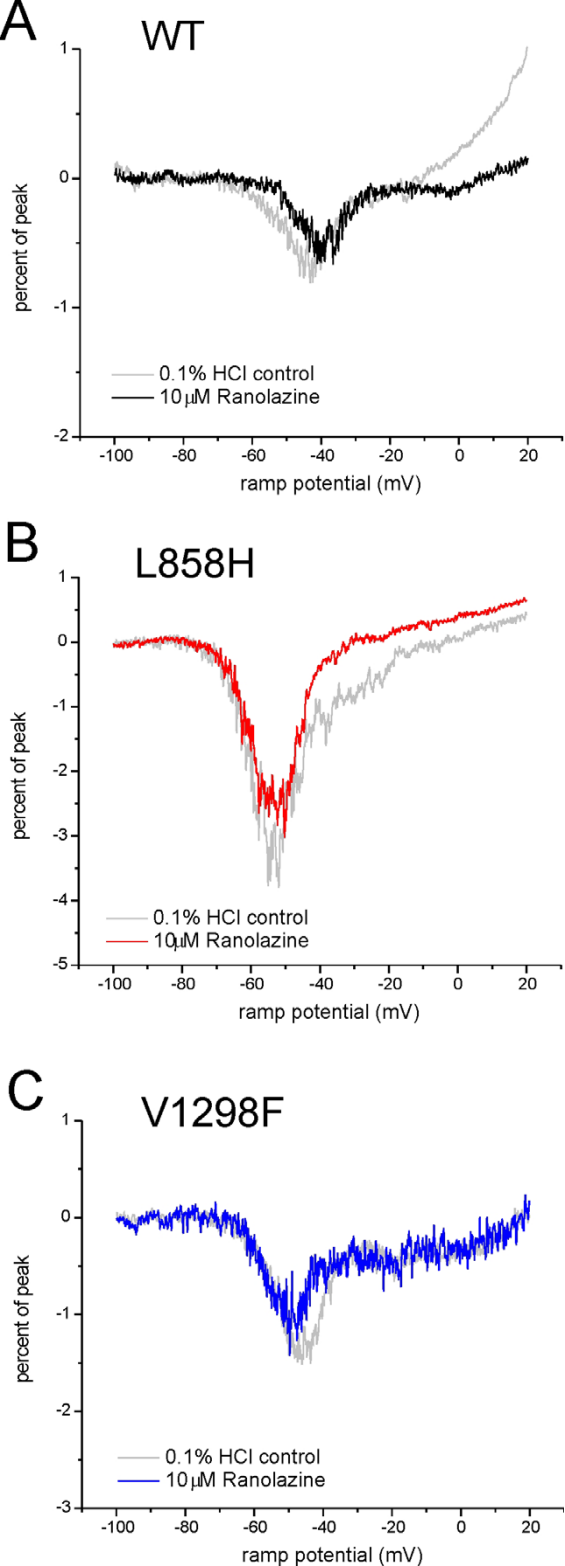
**Ranolazine block of ramp currents**. The response to slow gradual depolarization (ramp) stimulus protocol (-100 mV to +20 mV over 600 msec) from representative cells is normalized to the peak inward current for that cell as described in Methods. Separate cells were exposed to either 0.1% HCl vehicle control (grey line) or 10 μM ranolazine (bold line). In each panel the two traces are overlaid to compare the response to ranolazine. (A) HEK 293 cells expressing WT show a peak ramp response of 0.58 ± 0.15% and the voltage peaks at -43.0 ± 1.2 mV in the presence of 0.1% HCl vehicle control (n = 3). In the presence of 10 μM ranolazine (n = 4), the peak ramp response was 0.79 ± 0.22% and the voltage peaks at -48.0 ± 3.9 mV. (B) HEK 293 cells expressing, the IEM mutant L858H show a peak ramp response of 4.48 ± 1.13% and the voltage peaks at -55.8 ± 2.2 mV in the presence of 0.1% HCl vehicle control (n = 5). In the presence of 10 μM ranolazine (n = 4), the peak ramp response was 3.31 ± 0.60% and the voltage peaks at -54.1 ± 2.0 mV. (C) HEK 293 cells expressing the PEPD mutant V1298F show a peak ramp response of 1.05 ± 0.21% and the voltage peaks at -48.5 ± 2.2 mV in the presence of 0.1% HCl vehicle control (n = 8). In the presence of 10 μM ranolazine (n = 7), the peak ramp response was 0.84 ± 0.11% and the voltage peaks at -49.7 ± 2.7 mV.

Therapeutically achievable concentrations of ranolazine are lower (2-8 uM) than the reported IC_50 _for Na_v_1.7 channels in HEK 293 cells, and in cardiac cells are thought to work by differentially blocking the persistent current of Na_v_1.5 while sparing the peak current [4-6]. Because of slow closed-state inactivation, Na_v_1.7 channels respond to a slow voltage ramp depolarization with a current near resting potential of DRG neurons (ramp current), which is thought to boost natural weak stimuli [13]. In addition to the shifts in the voltage-dependence of activation or fast-inactivation of IEM and PEPD mutations, ramp currents are significantly bigger for IEM and PEPD mutant channels, compared to WT [2]. Thus, we examined whether ranolazine could differentially block the signature Na_v_1.7 ramp current. As shown in figure 4, 10 μM ranolazine did not significantly reduce peak inward ramp current in WT-, L858H-, or V1298F-expressing HEK 293 cells compared to cells exposed to vehicle control. There was no evidence for a differential block of ramp currents in L858H or V1298F mutant expressing cells compared to hNav1.7r-WT expressing cells.

**Figure 5:**
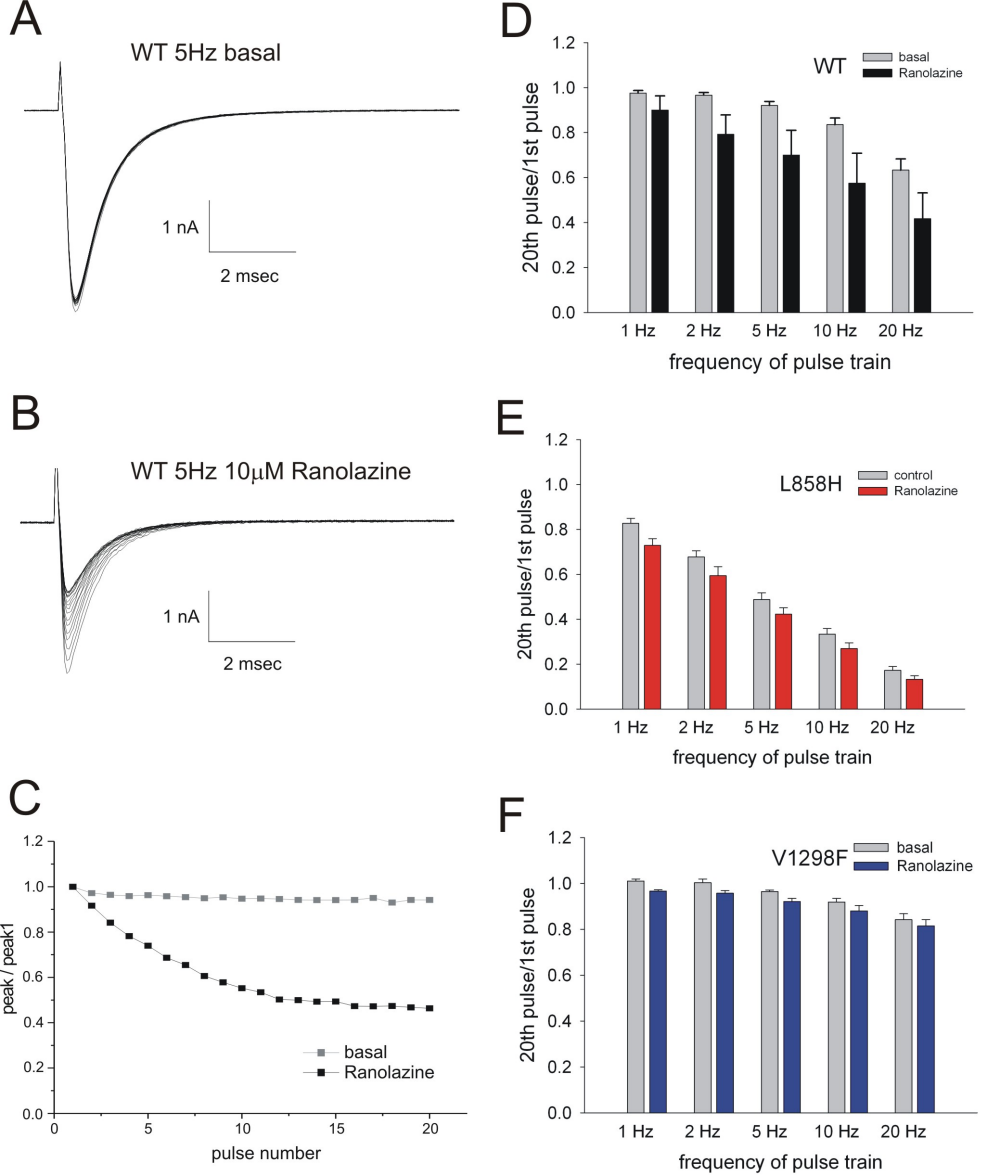
**Frequency-dependence of use-dependent block of Na_v_1**.7 currents by ranolazine. Trains of twenty 30 msec duration pulses to -10 mV from a holding potential of -120 mV were applied at five different frequencies and were performed both before and after exposure to 10 μM ranolazine. Example traces from a HEK 293 cell expressing WT channels are shown in panels A and B. The peak for each pulse is normalized to the peak of the first pulse, and the values are plotted in panel C. The use-dependent block, defined as the ratio of the peak from the 20^th ^pulse normalized to the peak of the first pulse, was determined for each cell and the averages are plotted with the basal responses shown in grey and the ranolazine responses shown in solid bars. In each panel the two datasets are shown to compare the response to ranolazine in cells expressing WT (panel D, n = 5), the IEM mutant L858H (panel E, n = 4), or the PEPD mutant V1298F (panel F, n = 7), channels.

Use-dependent block was examined to see whether either the L858H IEM mutant or the V1298F PEPD mutant showed a differential response to this protocol compared to WT channels. The example in Figure 5 shows the change in peak response to a train of 20 pulses applied to HEK 293 cells expressing WT channels at 5 Hz both before (Figure 5A) and after exposure to 10 μM ranolazine (Figure 5B). The graph in Figure 5C shows the relative block that occurs during the pulse train, obtained by dividing the peak of each pulse by the peak of the first pulse, for the data in panels 5A and 5B. In the absence of the drug, WT channels show use-dependence at frequencies greater than 5 Hz, suggesting that there is some accumulation of inactivated channels as the recovery period between pulses is shortened. After exposure to 10 μM ranolazine, there was a significant increase in the use-dependent reduction at all stimulation frequencies. For the L858H IEM mutant channels, (Figure 5E) there was notably more basal use-dependence at all stimulation frequencies. 10 μM ranolazine also caused a small but significant additional use-dependent response at all frequencies. The V1298F PEPD mutant channels, however, showed reduced basal use-dependence compared to WT channels while still showing the small but significant increase in response to 10 μM ranolazine (Figure 5F).

**Figure 6:**
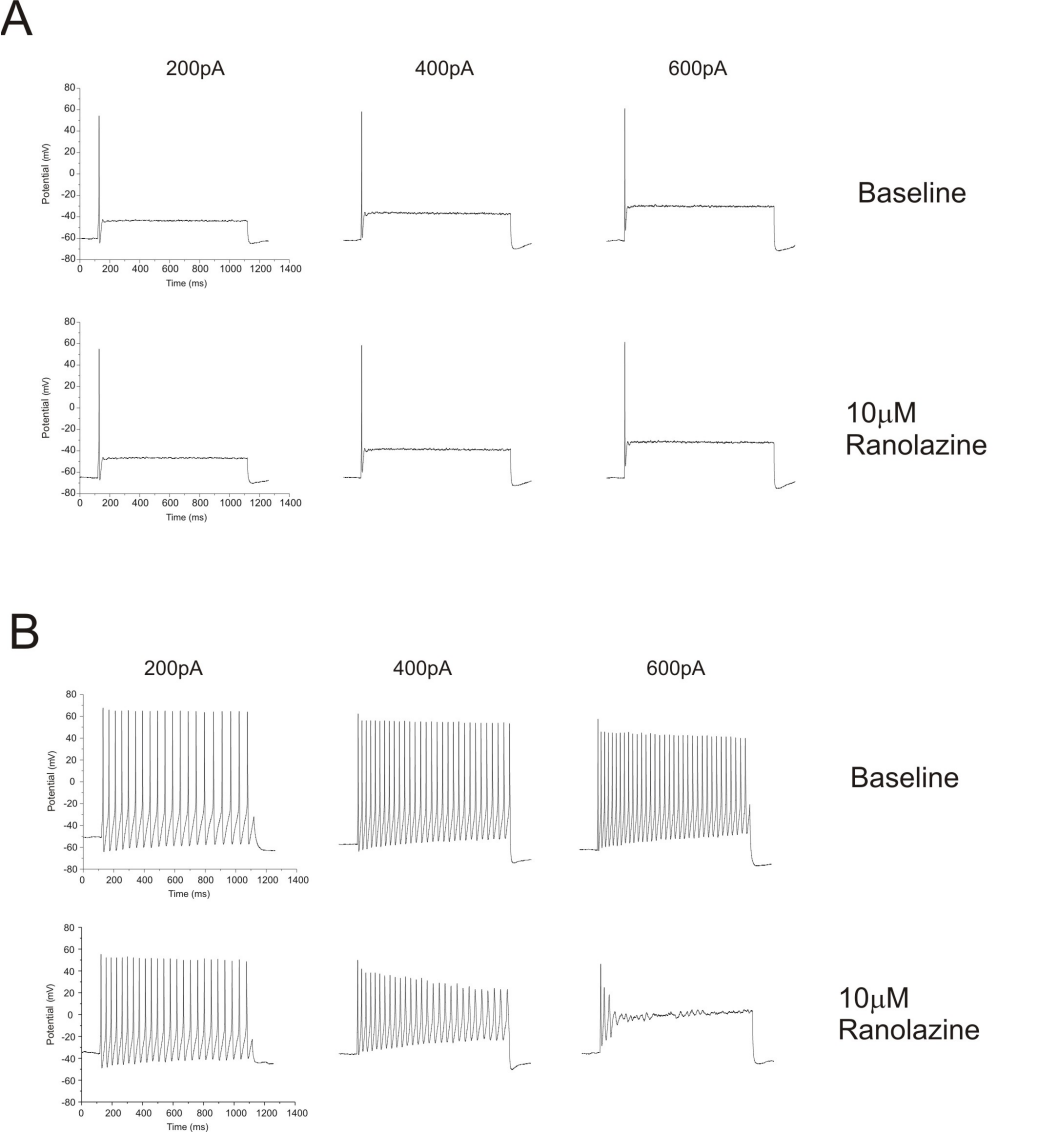
**Example responses to ranolazine from WT-expressing DRG neurons**. (A) Traces illustrating the response to 1 second duration current injections of 200pA, 400pA, and 600pA both before (upper row) and after (lower row) exposure to 10 μM ranolazine are shown from a DRG neuron transfected with hNav1.7-WT channels. This phenotype, showing low frequency firing, typically occurs in 5 of 6 WT-expressing DRG neurons chosen for recording. (B) Traces in the same format as panel A recorded from a high frequency firing WT-expressing DRG neuron.

**Figure 7:**
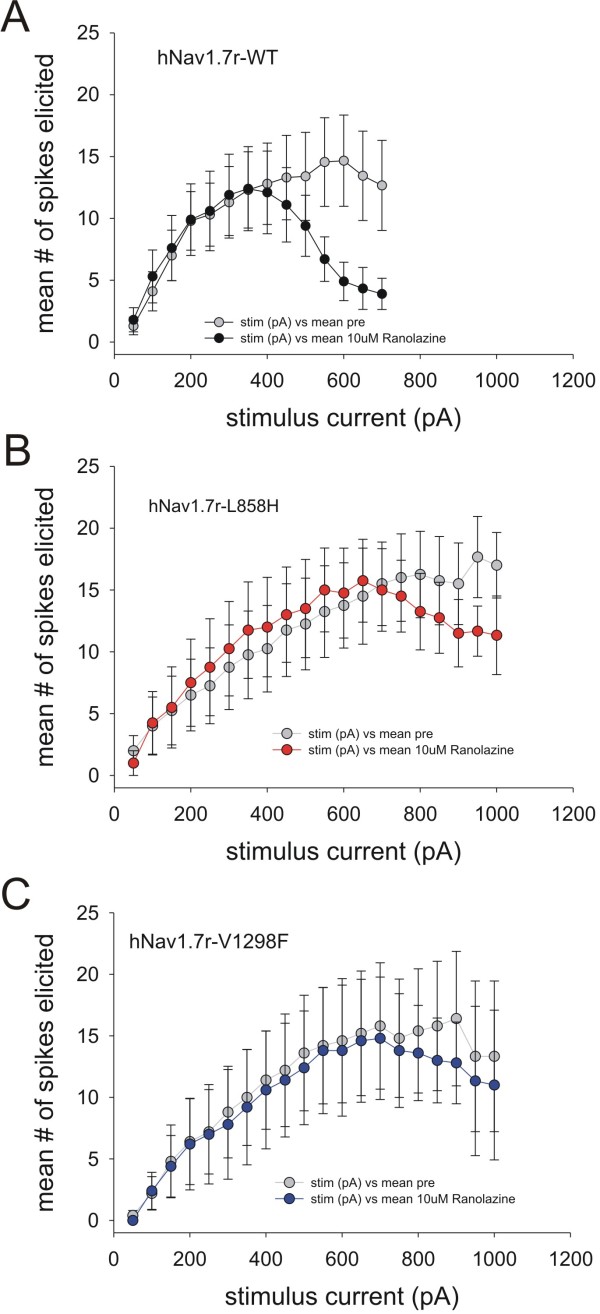
**Effect of ranolazine on the excitability of DRG neurons expressing WT, L858H or V1298F channels**. Action potentials were recorded in current-clamp mode from DRG neurons transfected with WT (panel A), the IEM mutant L858H (panel B), or the PEPD mutant V1298F (panel C), channels as described in the Methods section. The number of action potentials elicited during current injections of 1-sec duration ranging from 50 pA to 1000 pA in 50 pA increments are counted both before and then again after exposure to 10 μM ranolazine. In this figure, cells whose response did not exceed 5 spikes were removed to compare just the high firing cells. The average number of action potentials elicited at each current injection level are plotted before (grey) and after 10 μM ranolazine (solid) exposure. In each panel the two datasets are shown to compare the response to ranolazine in cells expressing WT (panel A, n = 10), the IEM mutant L858H (panel B, n = 4), or the PEPD mutant V1298F (panel C, n = 5), channels.

We directly tested whether ranolazine could reduce excitability of DRG neurons transfected with either WT or the hyperexcitability-inducing mutations L858H or V1298F. The majority of DRG neurons transfected with WT channels fire 1-2 action potentials in response to a one second depolarizing stimuli, while neurons transfected with Nav1.7 channels carrying IEM or PEPD mutations fire repetitively [14-18]. As shown in Figure 6 A, there was no obvious effect of 10 μM ranolazine on low frequency firing WT-expressing DRG neurons. In contrast, a few WT-expressing DRG neurons showed a high frequency firing phenotype and ranolazine clearly seemed to attenuate the response to the stronger depolarizing current stimuli.(Figure 6B). Thus, we selected neurons that fired multiple action potentials to study the effect of ranolazine on DRG neuron excitability. The averaged data shown in Figure 7 are compiled from the subset of neurons that fired at least five action potentials, recorded before ranolazine application in response to any current injection step, over the entire range of 50 to 1000 pA. The effect of 10 μM ranolazine treatment on DRG neurons transfected with WT is shown in Figure 7 A. Before ranolazine, the cells exhibited a smoothly increasing number of action potentials elicited in response to increasing current injections reaching an average of 14 spikes during the 1 second long stimulus. After ranolazine, there was no change in the threshold to first spike or in the number of spikes elicited. For DRG neurons transfected with WT Na_v_1.7 channels, the main effect of ranolazine was to attenuate the number of spikes elicited at the higher current stimulation range. The number of spikes elicited for current injections of 600 pA or greater was significantly reduced by 10 μM ranolazine. In contrast, there was no effect of ranolazine on the number of spikes elicited at any stimulus level on DRG neurons expressing either the L858H IEM mutant (Figure 7B) or the V1298F PEPD mutant (Figure 7C).

## Discussion

Ranolazine is a relatively new anti-anginal drug which preferentially blocks late but not peak Na_v_1.5 sodium currents at clinically-relevant concentrations [6], and has been shown to shorten the action potential duration in cardiac myocytes expressing an LQT5 Na_v_1.5 mutation but not wild type cardiac channels [6]. We evaluated ranolazine as a blocker of wild-type Na_v_1.7 channels and of mutant Na_v_1.7 channels that underlie two different neuropathic human pain disorders, IEM and PEPD, and assessed its ability to reduce excitability of DRG neurons that express WT or pain-causing mutant Na_v_1.7 channels. Using voltage-clamp we show here that ranolazine blocks WT and mutant Na_v_1.7 channels in a voltage-dependent manner, with greater block of inactivated channels, but with comparable effectiveness against WT and mutant Na_v_1.7 channels. We also show that ranolazine does not preferentially block ramp currents, compared to peak currents. Using current-clamp we show that ranolazine, at a clinically-relevant concentration, attenuates excitability of DRG neurons expressing WT but not the two mutant Na_v_1.7 channels examined here. These data suggest that ranolazine may be useful for treatment of some pain conditions in which up-regulated Na_v_1.7 expression, or modulation of this channel, contributes to DRG neuron hyperexcitability (see [11,12,19]).

The low affinity block of hNa_v_1.7 channels in the resting state (hyperpolarized holding potentials more negative than -100 mV) is in agreement with previously reported data [8,9], in which little block was observed by the highest drug concentration used. We have now extended this finding to two pain-linked mutations of hNa_v_1.7: the IEM disease-causing mutation L858H that exhibits a hyperpolarizing shift of the voltage-dependence of activation with no effect on fast-inactivation [20], and the PEPD disease-causing mutation V1298F that exhibits a depolarizing shift in the voltage-dependence of fast-inactivation with little effect on activation ([21,22]). Ranolazine appears to share a binding site with local anesthetics (LA) in sodium channels Na_v_1.4 [8] and Na_v_1.5 [6]. The reduced affinity for ranolazine to the closed-state of hNa_v_1.7 reported here and by Wang et al. (2008) suggests that ranolazine access to its site is occluded when the hNa_v_1.7 channels are hyperpolarized. Neither the L858H or V1298F mutant channels at rest (holding potential of -120 mV) show a reduced block by ranolazine, compared to WT channels (Table 1), suggesting no significant mutation-induced conformational change of the structure of the channel.

Our results show that the apparent affinity for ranolazine block of hNa_v_1.7 is voltage-dependent, with increasing effectiveness of channel block with depolarizing conditioning pulses. The conditioning pulse protocol used here utilized a 100 msec pulse to -120 mV to recover unblocked channels from fast-inactivation which will then be activated with a strong test depolarization pulse. The study by Wang et al. (2008) used 95 msec recovery pulse to -120 mV and reports similar IC_50 _values (59.9 ± 3.8 μM with Vcond = -70 mV) in close agreement with our study (34 μM with Vcond = -60 mV). In contrast, the study by Rajamani et al. (2008) utilized a 20 msec recovery pulse and reported a much lower IC_50 _value (3.25 μM with Vcond = -70 mV). We have previously reported that the time constant for repriming of hNa_v_1.7 channels at -120 mV is about 20 msec [13,23], so only about 63% of the channels available to recover will have done so with a 20 msec recovery pulse. Although the 100 msec recovery pulse is effective at differentiating slow-inactivation from fast-inactivation, the kinetics of ranolazine unbinding to closed (Vm = -120 mV) channels may affect the IC_50 _values reported here. The differences in the IC_50 _values between our data and previous reports might be explained by these differences in recording protocols.

Enhanced ranolazine block with more depolarized conditioning potentials suggests that the higher affinity interaction of ranolazine is with open or inactivated states of the Na_v_1.7 channel. Wang et al. (2008) used mutational ablation of the fast-inactivation gate to argue that ranolazine preferentially binds to the open state of hNa_v_1.7 channels and that the fast-inactivated conformation is not required for ranolazine block. At the conditioning potential of -60 mV, close to typical resting membrane potential of small DRG neurons [14-18,24,25], there was no apparent change in the effectiveness of ranolazine block for either of these mutant channels compared to wild-type channels. The apparent similarity of ranolazine block of WT or mutant Na_v_1.7 channels with either hyperpolarized activation and enhanced slow-inactivation (L858H [20]), or depolarized fast-inactivation and impaired slow-inactivation (V1298F [21,22]), suggest that the rate-limiting step for ranolazine-induced block of peak sodium currents may depend on the early transitions of the channels from the closed to the open state and less on the fully-activated or fully-inactivated channel states.

The ability of Na_v_1.7 to produce a current in response to small, slow depolarizations (ramp current) [13], thus boosting weak stimuli, contributes to the role of Na_v_1.7 as a threshold channel [26]. Most mutations that underlie IEM and PEPD increase amplitude of ramp currents of mutant Na_v_1.7 channels even when the peak currents were either unchanged or even reduced [2]. Because ranolazine is more effective at blocking late sodium current than peak current [4-6], we tested the hypothesis that Na_v_1.7 ramp currents may be preferentially blocked by ranolazine. We show here that ranolazine, at the clinically-relevant concentration of 10 μM, does not differentially block ramp currents produced by mutant Na_v_1.7 channels compared to WT. Together with our results showing similar IC_50 _of ranolazine block of WT and mutant Na_v_1.7 peak currents, these data suggests that ranolazine may not have a preferential effect in blocking mutant Na_v_1.7-induced DRG hyperexcitability.

Use-dependent loss of current in the absence of blockers suggests that there is progressive accumulation of channels into slow-inactivated states. The L858H mutant exhibits the most use-dependence in the absence of ranolazine (Figure. 5E) which is predicted by its enhanced slow-inactivation [20], while the V1298F mutant exhibited the least use-dependence in the absence of drug (Figure. 5F) consistent with the depolarized shift of fast- and slow-inactivation curves by this PEPD mutant [21,22]. The additional drug-induced use-dependence was small and constant over all stimulation frequencies for both the L858H and V1298F mutant channels. In contrast, ranolazine showed increased use-dependent block with higher frequency stimulation on WT channels (Figure 5D).

Ranolazine block of sodium channels in HEK 293 cells may not capture the full effect in native neurons. Therefore, we directly tested the effect of ranolazine on DRG neuron firing. Our data show a significant ranolazine-induced attenuation of neuronal excitability in DRG neurons expressing WT channels (Figure. 7A). The effect of ranolazine (at 10 μM) did not offset the hyperexcitability induced by the two Na_v_1.7 disease-causing mutants. Previously, we have shown that the presence of Na_v_1.8 is important for the manifestation of mutant Na_v_1.7 effects on neuronal excitability [18]. While ranolazine can block Na_v_1.8 channels with an IC_50 _similar to that of Na_v_1.7 [9], the effect on Na_v_1.8 is not enough to attenuate the mutant Na_v_1.7-induced neuronal hyperexcitability.

The reduction of firing in WT expressing DRG neurons only occurred with stronger current injections which would cause greater depolarization during the current injection. The combination of increasing ranolazine blocking effectiveness with depolarization and the use-dependent inhibition of Na_v_1.7 and other ion conductances in this select group of neurons might explain this attenuation of high firing rates. It is important to note that the cells that are transfected with WT channels on average fire at a lower frequency, compared to neurons that are transfected with mutant Nav1.7 channels [14-18,22,27]. In order to measure the effect of ranolazine on DRG neurons expressing WT channels, we selected WT-transfected neurons that fire at higher frequency compared to average transfected neurons, and it is reasonable to assume that these high-frequency firing neurons may express a different complement of ion channels than the average transfected neuron. Ranolazine, which is known to block other ion channels albeit with different kinetics compared to the block of late sodium currents [28], may block ion conductances that are differentially expressed in the WT-transfected high frequency-firing neurons reported here, leading to the differential effect on WT-transfected neurons compared to mutant-transfected neurons.

## Conclusions

Our data showing ranolazine block of high frequency firing of DRG neurons expressing WT hNa_v_1.7 channels suggest that ranolazine may provide relief from pain associated with hyperexcitability of DRG neurons expressing WT Nav1.7 channels. Indeed, ranolazine has been shown to ameliorate pain behavior in animal models of acquired neuropathic pain, although pain relief was short-lived, lasting only 30-90 min depending on oral or intraperitoneal delivery of the drug, and was more effective against cold-induced pain than mechanical allodynia [29]. Ranolazine appears to be a safe drug for human patients [30], and does not induce ataxic effects in animals [29], and its utility for neuropathic and inflammatory pain treatment merits further study.

## Materials and methods

### Voltage-clamp electrophysiology

The L858H (IEM, [20,31]) and V1298F (PEPD, [21,22,32]) mutations were introduced into a TTX-resistant (TTX-R) version of human Na_v_1.7 cDNA (hNa_v_1.7_R_, will be referred to as WT, hereinafter) using QuickChange XL site-directed mutagenesis (Stratagene, La Jolla, CA). Transfected HEK 293 cells, grown under standard culture conditions (5% CO_2_, 37°C) in Dulbecco's modified Eagle's medium supplemented with 10% fetal bovine serum, were treated with G418 for several weeks to derive stable cell lines that express the mutant channels as described previously [33,34]. Use of stable cell lines reduces variability in terms of current expression, and lends itself to pharmacological profiling on an automated patch-clamp platform, PatchXpress (Molecular Dynamics, Union City, CA).

Whole-cell voltage-clamp recordings were performed on isolated HEK 293 cells stably expressing WT or L858H or V1298F mutant channels at room temperature (~21°C). Electrodes were pulled from 1.6 mm O.D. borosilicate glass micropipettes (WPI, Sarasota, FL) and had a resistance of 1-2 MΩ when filled with pipette solution, which contained (in mM): 140 CsF, 10 NaCl, 10 HEPES, 1 EGTA (pH 7.3 with CsOH, adjusted to 320 mOsm with dextrose). The extracellular solution contained (in mM): 140 NaCl, 3 KCl, 1 MgCl_2_, 1 CaCl_2_, 10 HEPES (pH 7.3 with NaOH, adjusted to 320 mOsm with dextrose). Voltage-clamp currents were recorded 5 min after establishing whole-cell configuration on an Axopatch 200B amplifier (Molecular Devices, Sunnyvale, CA) and stored via a Digidata 1440A A/D converter (Molecular Devices) at an acquisition rate of 50 kHz with a lowpass Bessel filter setting of 10 kHz. Voltage errors were minimized with 80-95% series resistance compensation, and only cells with <3 mV voltage error after compensation were included for analysis. When appropriate, linear leak currents and capacitance artifacts were subtracted out using the P/N method provided by Clampex (Molecular Devices) acquisition software. Clampfit (Molecular Devices) and Origin (Microcal Software, Northhampton, MA) were used for data analysis. Data are expressed as means ± standard error (SEM). Statistical significance was determined by Student's t-test. For datasets in which data were collected from the same cell before and then after exposure to ranolazine (use-dependence in voltage-clamp, action potential firing in current-clamp) a paired Student's t-test was performed.

The slow ramp protocol smoothly varies the command potential from -100 mV to 20 mV over 600 msec (0.2 mV/msec). At this ramp rate fast Na-channels such as Nav1.1, Nav1.2 and Nav1.6 undergo complete closed-state inactivation and do not produce an inward current in response to this stimulation protocol. hNav1.7, however, has slowed closed-state inactivation kinetics and can still exhibit a signature inward current during the slow ramp [13]. To compare between cells, the ramp response is scaled by the scalar value of the peak activation I-V response (e.g. for a cell with -1500pA peak current, its slow ramp will be divided by 1500). This maintains the convention that inward currents are shown as downward deflections in figures. The magnitude of the peak as well as the voltage at peak are determined for each cell. These values are averaged by channel type and compared using Student's t-test.

For voltage-clamp studies examining the dose-response to ranolazine treatment, stock solutions of ranolazine (Supplied by Gilead Sciences Inc.) with a concentration of 10 mM were prepared in 0.1N HCl aqueous solution. Studies were performed in extracellular solution containing 0.001N HCl (control) or working stock concentrations of ranolazine prepared freshly in 0.1N HCl and then diluted 1:100 in extracellular bath solution to give the indicated drug final concentrations. The recordings were obtained using the PatchXpress (Molecular Devices) automated patch-clamp system. The effect of ranolazine on the WT and L858H and V1298F mutant channels was tested from the resting and inactivated states. To determine the voltage-dependence of ranolazine block, the voltage clamp protocol was as follows: the membrane potential was held at conditioning potentials that varied from -120 mV (resting block) to -40 mV (inactivated block) for 10 sec to equilibrate Na_v_1.7 channels in the presence of vehicle (HCl) or ranolazine in the bath solution; the membrane potential was then pulsed to -120 mV for 100 msec to allow channels not bound by drug to recover from fast-inactivation, and then given a 20 msec test pulse to 0 mV to elicit current from available channels. To control for time-dependent changes in current expression, independent cells were exposed to only one test concentration of ranolazine or vehicle at a consistent time after initiation of whole-cell recording. The response was normalized to the currents elicited from the same protocols applied just before compound addition, and data expressed as fraction of current remaining. These normalized responses from 3-8 independent cells for each ranolazine concentration were averaged and plotted to make a dose-response curve. The half maximal inhibitory concentration (IC_50_) of ranolazine for WT and mutant channels were obtained by fitting the data at each conditioning potential using the *Dose Response *function in Origin. Statistical comparisons of these fits were performed using the *compare data *function in Origin. Differences are considered significant when p < 0.05.

### Dorsal root ganglia neuron isolation and transfection

DRG from Sprague Dawley rat pups (P1-P5) were isolated and then cultured as previously described [35]. Either WT or mutant (L858H or V1298F) channels were transiently transfected into DRG neurons, along with enhanced-GFP, by electroporation with a Nucleofector II (Amaxa, Gaithersburg, MD) using Rat Neuron Nucleofector Solution and program G-013 as previously described [35]. The ratio of sodium channel to GFP constructs was 5:1. The transfected neurons were allowed to recover for 5 minutes at 37°C in 0.5 ml of Ca^2+^-free DMEM containing 10% fetal calf serum. The cell suspension was then diluted with DRG media containing 1.5 mg/ml bovine serum albumin and 1.5 mg/ml trypsin inhibitor, 80 μl was plated on 12 mm circular poly-D-lysine/laminin precoated coverslips (BD Biosciences, Bedford, MA) and the cells incubated at 37°C in 5% CO_2 _for 30 min. DRG media (1 ml/well), supplemented with 50 ng/ml each of mNGF (Alomone Labs, Jerusalem, Israel) and GDNF (Peprotec, Rocky Hill, NJ), was then added and the cells maintained at 37°C in a 5% CO2 incubator.

### Current-clamp electrophysiology

Whole-cell current-clamp recordings were performed using the Axopatch 200B amplifier, digitized using the Digidata 1440A interface and controlled using pCLAMP software. The bath solution for current clamp recordings contained (in mM): 140 NaCl, 3 KCl, 2 MgCl_2_, 2 CaCl_2_, and 10 HEPES, pH 7.3 with NaOH (adjusted to 315 mOsm with dextrose). The pipette solution contained (in mM): 140 KCl, 0.5 EGTA, 5 HEPES, and 3 Mg-ATP, pH 7.3 with KOH (adjusted to 300 mOsm with dextrose). The junction potential between these two solutions given by JPcalc was 5 mV but no correction was applied for current-clamp experiments. Recordings were performed on transfected presumptive nociceptive neurons based on the morphology of small diameter (20-25 μm) round cell bodies that also exhibited green fluorescence. All recordings were performed between 20 hr-50 hr post-transfection. Cover slips were transferred to a perfusable chamber (Warner Instruments, Hamden, CT) and all recordings were initiated within an hour. Whole-cell configuration was obtained in voltage-clamp mode before proceeding to the current-clamp recording mode. Cells with stable (< 10% variation) resting membrane potentials (RMPs) more negative than -35 mV and overshooting action potentials (>85 mV RMP to peak) were used for further data collection. Action potential frequency was determined by quantifying the number of action potentials elicited in response to depolarizing current injections of 1 second duration.

## Competing interests

The authors declare that they have no competing interests.

## Authors' contributions

ME designed and performed electrophysiological experiments, analyzed data and drafted the paper. SDD-H. and SGW. conceived and supervised the project and edited the manuscript. All authors contributed to data interpretation, have read and approved the final manuscript.
